# A multisession evaluation of an adaptive competitive arm rehabilitation game

**DOI:** 10.1186/s12984-017-0336-9

**Published:** 2017-12-06

**Authors:** Maja Goršič, Imre Cikajlo, Nika Goljar, Domen Novak

**Affiliations:** 1 Department of Electrical and Computer Engineering, University of Wyoming, 1000 E University Avenue, Laramie, WY 82071 USA; 2 University Rehabilitation Institute of the Republic of Slovenia, Linhartova 51, SI-1000 Ljubljana, Slovenia; 3 University of Nova Gorica, School of Engineering and Management, Vipavska 13, SI-5000 Nova Gorica, Slovenia

**Keywords:** Arm rehabilitation, Virtual reality, Multiplayer games, Dynamic difficulty adaptation, Interpersonal rehabilitation games, Social interaction, Motivation, Exercise intensity

## Abstract

**Background:**

People with neurological injuries such as stroke should exercise frequently and intensely to regain their motor abilities, but are generally hindered by lack of motivation. One way to increase motivation in rehabilitation is through competitive exercises, but such exercises have only been tested in single brief sessions and usually did not adapt difficulty to the patient’s abilities.

**Methods:**

We designed a competitive arm rehabilitation game for two players that dynamically adapts its difficulty to both players’ abilities. This game was evaluated by two participant groups: 15 participants with chronic arm impairment who exercised at home with an unimpaired friend or relative, and 20 participants in the acute or subacute phase of stroke who exercised in pairs (10 pairs) at a rehabilitation clinic. All participants first played the game against their human opponent for 3 sessions, then played alone (against a computer opponent) in the final, fourth session. In all sessions, participants’ subjective experiences were assessed with the Intrinsic Motivation Inventory questionnaire while exercise intensity was measured using inertial sensors built into the rehabilitation device. After the fourth session, a final brief questionnaire was used to compare competition and exercising alone.

**Results:**

Participants who played against an unimpaired friend or relative at home tended to prefer competition (only 1 preferred exercising alone), and exhibited higher enjoyment and exercise intensity when competing (first three sessions) than when exercising alone (last session).

Participants who played against each other in the clinic, however, did not exhibit significant differences between competition and exercising alone. For both groups, there was no difference in enjoyment or exercise intensity between the first three sessions, indicating no negative effects of habituation or novelty.

**Conclusions:**

Competitive exercises have high potential for unsupervised home rehabilitation, as they improve enjoyment and exercise intensity compared to exercising alone. Such exercises could thus improve rehabilitation outcome, but this needs to be tested in long-term clinical trials. It is not clear why participants who competed against each other at the clinic did not exhibit any advantages of competition, and further studies are needed to determine how different factors (environment, nature of opponent etc.) influence patients’ experiences with competitive exercises.

**Trial registration:**

The study is not a clinical trial. While human subjects are involved, they do not participate in a full rehabilitation intervention, and no health outcomes are examined.

**Electronic supplementary material:**

The online version of this article (10.1186/s12984-017-0336-9) contains supplementary material, which is available to authorized users.

## Background

### Rehabilitation games

Stroke is a leading cause of disability, with 795,000 new or recurrent strokes per year in the United States alone [1]. 88% of survivors experience motor function impairment and thus require rehabilitation to regain their movement abilities [2]. However, even top hospitals devote only an hour per day to motor rehabilitation [3], and exercise intensity is usually too low for optimal rehabilitation outcome [4]. Patients are thus expected to exercise independently at home after leaving the clinic to fully regain their abilities, but frequently do not exercise frequently or intensely enough. For example, one study found that only 30% of unsupervised patients comply with prescribed home rehabilitation regimens [5]. Another home rehabilitation study found that patients average around 1.5 h of exercise per week [6], while clinical studies involve at least 3 h of exercise per week [7, 8]. To improve home rehabilitation, it is therefore critical to increase the frequency and intensity of exercise.

One key reason for poor compliance in home rehabilitation is lack of motivation, which is an important predictor of rehabilitation outcome [9, 10]. While the definition of motivation in rehabilitation is blurry, it is generally agreed to involve a willingness to actively engage in exercise [11, 12]. To improve engagement, researchers have thus developed numerous rehabilitation games that try to both ensure high enjoyment (using, e.g., meaningful goals, in-game rewards and entertaining graphics [12–15]) and provide an appropriate exercise intensity via automated difficulty adaptation [12, 14, 16]. The games are controlled using motion tracking hardware such as the Microsoft Kinect or even with rehabilitation robots that provide limb support in addition to motion tracking. However, recent reviews have emphasized that such games are not yet sufficiently engaging for all patients [17, 18]. Therefore, additional rehabilitation game development and validation is necessary to improve patient engagement.

### Competitive and cooperative motor rehabilitation games

One way to improve engagement is through competitive or cooperative games, which allow patients to interact with another person while exercising. Such games are highly motivational when used for entertainment [19, 20] and weight loss [21]. Particularly in games for weight loss, competition and cooperation increase enjoyment and exercise intensity compared to exercising alone [21, 22], which would also be very useful for motor rehabilitation.

Several single-session studies with stroke survivors have found that both competition and cooperation can increase patient engagement compared to exercising alone [23–25]. Particularly competition was found to be promising, as it results in higher exercise intensity than cooperation or exercising alone [24]. However, these studies only examined engagement in single brief sessions. As engagement may decrease over multiple sessions as patients become familiar with the game [16], competitive games should be examined over multiple sessions to determine whether their benefits are maintained for a longer time period. This was preliminarily done by Maier et al. [26], who observed high enjoyment and exercise intensity over three sessions, but in only two stroke survivors and with no control condition (i.e. only competitive games). A larger multisession study is thus necessary to obtain additional insights about the effects of competition in motor rehabilitation.

To keep patients engaged over multiple sessions, game difficulty should be dynamically adapted over time. Such difficulty adaptation is a crucial element of most rehabilitation games [12, 14], but is more challenging in competitive games, as difficulty must be adapted for two players that may have different motor abilities. As previous studies had not yet developed appropriate adaptation algorithms, they either used a constant difficulty level throughout the session [23, 24, 27] or adapted difficulty to be the same for both players, without accounting for potential skill differences [25]. While several advanced adaptation algorithms have now been proposed for competitive games, they have been evaluated only with unimpaired participants [28, 29] or with very small numbers of patients [26, 30, 31]. Still, these evaluations have shown that difficulty adaptation has the potential to make a major contribution to competitive exercises.

### Contribution of paper

Our paper describes a four-session evaluation of a competitive arm rehabilitation game enhanced with the difficulty adaptation strategy from our previous work [30]. This evaluation was performed with participants who had chronic arm impairment and competed either with unimpaired friends/relatives or with other impaired participants. Our research questions were:

- In a four-session exercise protocol involving competitive rehabilitation games, do enjoyment and exercise intensity decrease from session to session?
- In a four-session exercise protocol, does competition result in higher enjoyment and exercise intensity than exercising alone?
- Do effects of competition depend on whether participants are competing against an unimpaired friend/relative or against another person with motor impairment?

Both enjoyment and exercise intensity are critical aspects of patient engagement and are correlated with rehabilitation outcome [9, 10, 32–34], so a long-term increase in both can be expected to lead to higher rehabilitation outcome. While our study is limited to four sessions and thus cannot guarantee that any benefits would persist over a full clinical trial, it provides a better estimate of potential benefits than previous single-session studies. For example, our recent study of rehabilitation games played by a single person showed that at least three sessions are needed to capture negative effects of habituation on engagement [35].

## Methods

### Participants

Data collection was carried out between September 2016 and May 2017. Inclusion/exclusion criteria were: hemiparesis due to a neurological injury; age 18–80 years; no cognitive impairment that would prevent participants from following study instructions or reporting problems; no history of seizures; no history of severe, progressive or current unstable medical conditions; no profound atrophy of arm muscles; no significant vision problems (e.g. unilateral neglect, visual field loss); and no significant spasticity (defined as inability to hold the rehabilitation device). Lack of ability to move the paretic limb was not an exclusion criterion, as the rehabilitation device (Hardware and Software section) allows participants to use their unimpaired arm to assist the paretic one.

Two participant groups were recruited:

- 16 participants with chronic arm impairment who participated together with an unimpaired friend or relative. One dropped out after the first session, resulting in 15 valid impaired participants (9 females, 6 males, 52.7 ± 13.7 years old). Three exercised with a friend, 7 with their spouse, 3 with their adult son or daughter, and 2 with their brother. The impairment was due to ischemic stroke (9 participants), hemorrhagic stroke (2), an ischemic stroke followed by a hemorrhagic one (1), traumatic brain injury (1), or cerebral palsy (2 participants). Participants with cerebral palsy were diagnosed with it shortly after birth; for the other participants, the injury occurred 7.4 ± 4.9 years prior to study participation. Ten had an impaired right arm while 5 had an impaired left arm. They were recruited via support groups and mailing lists in Wyoming, Colorado and Slovenia, and screened for inclusion by co-author DN. This group had an additional inclusion criterion: the injury must have occurred at least 6 months prior to study participation.
- 20 participants in the acute or subacute phase of stroke who were undergoing inpatient rehabilitation at the University Rehabilitation Institute of the Republic of Slovenia (5 females, 15 males, 57.8 ± 11.7 years old). They performed the exercises in pairs, resulting in 10 participant pairs. The stroke was either ischemic (16 participants) or hemorrhagic (4 participants) and occurred 4.6 ± 3.3 months prior to study participation. Eleven had an impaired right arm while 9 had an impaired left arm. They were screened for inclusion by co-author NG.

Participants in the first group performed the competitive exercises at their home or at another familiar location (e.g. community center), and will thus be referred to as the “home” group. Participants in the second group performed the exercises at a rehabilitation clinic, and will be referred to as the “clinical” group.

The degree of arm impairment was tested with the Box and Block test (BBT) of manual dexterity [36] at the beginning of the first session. The test assesses the ability to perform reaching and grasping movements, with a score of zero indicating no ability and a score above 50–60 indicating normal arm function. In the home group, the BBT score was 24.0 ± 18.9 for the impaired arm. In the clinical group, the BBT score was 29.1 ± 19.4 for the impaired arm and 53.7 ± 10.7 for the unimpaired arm. Additionally, participants in the clinical group were tested at the initial screening with the Mini-Mental State Examination (MMSE) [37], which assesses the severity of cognitive impairment and has a maximum (unimpaired) score of 30. Their MMSE score was 28.2 ± 2.0, with the lowest being 24. The MMSE was not used as an inclusion criterion.

### Hardware and software

#### Rehabilitation device

**Fig. 1:**
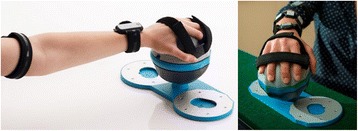
The Bimeo arm rehabilitation system in the wrist and forearm training configuration. Inertial sensors are attached to the upper arm, attached to the forearm, and integrated in the sphere that supports the hand

All impaired participants used the Bimeo arm rehabilitation system (Kinestica d.o.o., Slovenia) to play the rehabilitation game. The system consists of three inertial measurement units (IMUs) placed on the upper arm, on the forearm, and inside a handheld module that has a spherical shape and sits on a table (Fig. 1). The system tracks arm joint angles with an accuracy of approximately 2° in normal conditions [38, 39]. To play the rehabilitation game, the participant tilts the sphere left or right, with the required range of motion being 20° left and 20° right from the center position. This same setup was used in our previous research [24, 30].

The Bimeo was attached to the participant’s impaired arm by the experimenter at the beginning of all sessions. Participants were asked to hold the handheld module with only their impaired arm if possible, but were allowed to hold it with both hands if they had insufficient strength or mobility in the impaired arm. This is an intentional feature of the Bimeo, which is designed to allow hemiparetic users to support an impaired arm with the unimpaired one similarly to how rehabilitation robots provide assistance to an impaired arm. This approach was successfully used in our previous studies [24, 30].

In the home group, the unimpaired participant played the competitive game by tilting a Logitech joystick left and right from the center position. This approach was also used in our previous research [24, 30].

#### Adaptive competitive arm rehabilitation game

**Fig. 2:**
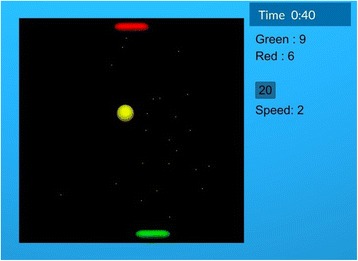
Pong game screenshot. The bottom and top paddles are controlled by the two participants. The current game duration, score, ball speed and time until the next automated difficulty adaptation are shown on the right side of the playing field. Paddles in the figure are at their default (initial) size

The competitive arm rehabilitation game is a modified version of the classic Pong game (Fig. 2). Each participant controls a paddle near the top or bottom of the screen and moves it left or right by tilting their Bimeo or joystick. A ball bounces around the game field, and each participant’s goal is to intercept the ball so that it does not pass their paddle. If the ball passes a player’s paddle and reaches the top or bottom of the screen, the opponent scores a point.

A basic version of the competitive game (without difficulty adaptation) was used in our previous work [24, 30]. In this basic version, the paddle width was 15.5% of the width of the game field and the ball speed was such that the ball required 1.3 s to cross the field if travelling directly across it (not at an angle). These settings were originally chosen so that they represented a moderate challenge for most stroke survivors. For the current study, we expanded the game with a combination of manual and automatic difficulty adaptation. The game starts at the basic difficulty, which is automatically adapted every 60 s as follows:

- If score has increased by at least 5 points for both participants in the last 60 s (indicating that both participants have been unable to intercept the ball), the ball speed decreases by 25% of the default speed value (e.g. from 75% to 50% of the default). As ball speed affects both participants, this decreases the difficulty for both participants.
- If score has increased by fewer than 5 points for both participants in the last 60 s (indicating that both participants have been successfully intercepting the ball), the ball speed increases by 25% of the default value (e.g. from 125% to 150% of the default).
- If score has increased by at least 5 points for one participant and fewer than 5 for the other (indicating that one participant has done well but the other has not), the size of the paddle decreases by 3.9% of the width of the game field (e.g. from 15.5% to 11.6% of the width of the field) for the participant who is performing well and increases by the same percentage (e.g. from 15.5% to 19.4%) for the participant who is not performing well. As paddle size affects each participant individually (a smaller paddle makes it harder to intercept the ball), this rule increases the game difficulty for the participant who is doing well and decreases it for the participant who is not doing well.

At any time, participants can also manually increase or decrease the ball speed or the size of each paddle by verbally telling the experimenter what they want to do. When a manual change is made, the game will wait 60 s to make the next automated change, and will not reverse the last manual change made – for example, if players manually increase ball speed and their performance is then poor, the game will increase paddle size for both players instead of decreasing the ball speed. Such automatic and manual adaptation was validated in our previous single-session study [30].

### Study protocol

Participants took part in four exercise sessions that were conducted within 1 week (clinical group, who were inpatients at the University Rehabilitation Institute) or 2 weeks (home group, who often had jobs and lived up to 1.5 h away from our institution, making scheduling more difficult). At the beginning of the first session, the study protocol was explained, and participants signed an informed consent form. A short game tutorial was given, and possible manual difficulty changes were demonstrated. Furthermore, a pre-game questionnaire and personality questionnaire were administered (see next section).

**Fig. 3:**
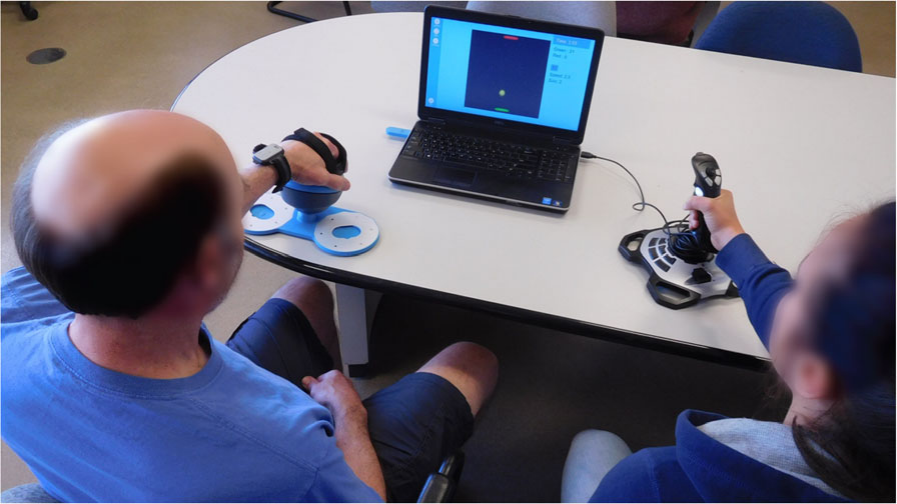
Experimental setup for the home group. An impaired participant and their unimpaired partner play the competitive Pong game (displayed on a screen in front of them) using a Bimeo and a joystick

The experimental setup is shown in Fig. 3 for the home group; the clinical group used a similar setup, but with two Bimeo devices (rather than one Bimeo and one joystick). In the first three sessions, both participants in the pair played the competitive game against each other for at least 10 min. A timer on the screen showed the game duration, and participants were told that they could stop playing whenever they wished once 10 min had passed. The experimenter stopped the exercise as soon as one participant requested to stop, but did not encourage participants to stop or keep exercising. At the end of each session, the Intrinsic Motivation Inventory (IMI) (see next section) was administered.

The last (fourth) session involved the same game, but with a computer-controlled opponent instead of the human opponent – in other words, a single-player (SP) session. The computer-controlled opponent exhibited the same behavior as in our previous study [24]. At the end of the session, the IMI as well as a final overall experience questionnaire (see next section) were administered. This final session served as a comparison of whether exercising alone would be less motivating than exercising with a human opponent (first three sessions).

### Subjective measures

All questionnaires are provided in full in Additional file 1, and are briefly summarized here.

#### Pre-game questionnaire

The pre-game questionnaire asked participants about their age, gender, how often they play computer games, how difficult they prefer games to be (7-point Likert item), and how much they enjoy competing with other people (7-point Likert item). A similar questionnaire was used in our previous studies on game difficulty adaptation [30, 40].

#### Personality

The Ten Item Personality Inventory questionnaire [41] measures 5 parameters: extraversion, agreeableness, conscientiousness, emotional stability and openness to experiences, each using two 7-point Likert items.

#### Subjective experience in each session

The Intrinsic Motivation Inventory (IMI) measures four aspects of engagement: enjoyment/interest, effort/importance, perceived competence and pressure/tension. While there are many versions of the IMI [42], we used one that includes two 7-point Likert items for each parameter. Each parameter thus has a minimum of 2 and maximum of 14. This version was used in our previous study and gave similar results to a longer 20-item IMI [24].

#### Overall subjective experience

The overall experience questionnaire asks participants to compare two game conditions: playing with someone else and playing alone. It includes three questions: “Which of the two game conditions did you prefer?” (5 possible answers: strongly/weakly preferred playing alone, no preference, weakly/strongly preferred playing with someone else), “Which of the two game conditions was more fun?” (7 possible answers: playing alone was slightly/moderately/much more fun, both were equally fun, playing with someone else was slightly/moderately/much more fun), and “In which of the two game conditions did you feel more tension?” (possible answers as for previous question, but with ‘tense’ instead of ‘fun’). A similar questionnaire was used in our previous studies on game adaptation [40, 30].

#### Conversation level

At the end of each session, the experimenter estimated the degree to which the participants talked to each other while playing the game. This was done on a 4-point scale where 0 represented essentially no conversation, 1 represented occasional conversation, 2 represented frequent conversation, and 3 represented essentially uninterrupted conversation throughout the session. The experimenter made this estimate without consulting the participants, and we acknowledge that it was likely biased to some degree. Nonetheless, it served as a quick and simple measure, and was inspired by our previous qualitative observations that pairs who talked frequently during the game tended to report higher enjoyment [24].

### Objective measures

#### Measurement of exercise intensity

The root-mean-square (RMS) of the angular velocity of the hand was calculated in the roll axis (which is used to control the competitive game) using measurements from the IMUs for all impaired participants. This RMS value is a good indicator of exercise intensity in arm rehabilitation [43] and was also used in our previous research [24, 30].

#### Duration

As our previous studies on cognitive rehabilitation indicate that highly engaged participants are likely to play longer [35], the duration of each session was logged as a secondary, objective measure of engagement.

#### Other game parameters

The speed of the ball, size of both paddles and score of both players were logged throughout the game.

### Data analysis

Analyses were conducted separately for the home group and the clinical group. This is because we expected (based on our previous work [23, 24]) that the two groups would have different experiences. To identify systematic differences between the two groups, Mann-Whitney rank sum tests were used to compare the groups’ five personality factors and answers to the pre-game questionnaire. In the clinical group, all 20 participants were treated as independent for purposes of analysis, though they are not truly independent – each participant’s experience depends on the other participant in the pair.

For each group, one-way repeated-measures analyses of variance (ANOVA) followed by post-hoc Holm-Sidak tests were conducted for IMI parameters, exercise intensity and session duration to identify differences between the four sessions. When normality requirements for ANOVA were not met, a Friedman test (one-way repeated-measures ANOVA on ranks) followed by post-hoc Tukey tests was conducted instead. For the parametric ANOVA, effect size is reported as partial eta-squared (η^2^); for the Friedman test, χ^2^ is reported instead.

To identify the effects of participants’ characteristics on enjoyment of competition, Spearman correlation coefficients (ρ) were calculated between each personality score, results of the pre-game questionnaire, and the overall experience questionnaire (30 correlations, as we did not calculate correlations between variables from the same questionnaire). To allow calculation of correlations, answers to the overall experience questionnaire were converted into numerical values. No correction for multiple correlation calculations was performed.

Finally, to identify the effect of conversation between participants on enjoyment of competition, Spearman correlation coefficients were calculated between the conversation level (as rated by the experimenter) and results of the IMI in each session separately (16 correlations). Furthermore, correlation coefficients were calculated between the conversation level in the first session and results of the overall experience questionnaire (3 correlations). Levels of conversation from only the first session were used since most participants’ conversation level was similar in all three sessions.

## Results

Results were analyzed separately for the home group and the clinical group. The two groups did not differ with regard to personality factors, though the difference in openness to experiences approached significance (*p* = 0.080, U = 86.5). The groups differed with regard to the pre-game questionnaire: participants in the home group played computer games more frequently (*p* = 0.001, U = 57.5) and preferred more difficult games (*p* = 0.028, U = 58.5).

### Home rehabilitation group

The home rehabilitation group consisted of 15 impaired participants who exercised together with unimpaired friends or relatives. One participant did not complete the fourth session since they became unavailable due to unrelated personal matters, but their data from the first three sessions were included in the analysis. Unless stated otherwise, all results are given for impaired participants; results from unimpaired partners are presented in the last subsection.

#### Intrinsic motivation inventory

**Fig. 4:**
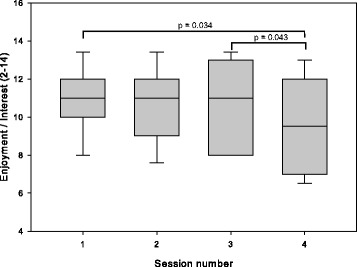
Enjoyment/interest measured using the Intrinsic Motivation Inventory for participants in the home group. In sessions 1–3, participants exercised with an unimpaired partner; in session 4, they exercised alone

For enjoyment/interest, ANOVA found an effect of session (*p* = 0.023, η^2^ = 0.21). Post-hoc tests found that enjoyment/interest was lower in the 4th (SP) session than in the first session and the third session, as illustrated in Fig. 4. The difference between the 2nd and 4th session was not significant (*p* = 0.16).

**Fig. 5:**
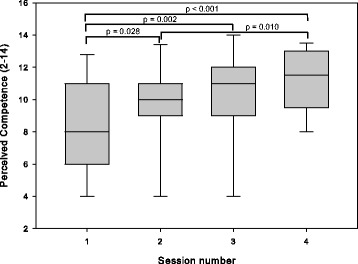
Perceived competence measured using the Intrinsic Motivation Inventory for participants in the home group. In sessions 1–3, participants exercised with an unimpaired partner; in session 4, they exercised alone

For perceived competence, ANOVA found an effect of session (*p* < 0.001, η^2^ = 0.46). Post-hoc tests found that competence tended to increase over time, as illustrated in Fig. 5.

ANOVA found no differences between sessions for effort/importance (*p* = 0.48, η^2^ = 0.06) or pressure/tension (*p* = 0.47, η^2^ = 0.07).

#### Overall subjective experience

**Fig. 6:**
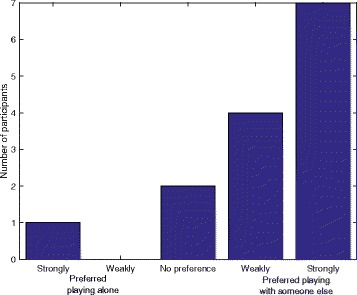
Responses of participants in the home group when asked if they preferred playing alone or with someone else

**Fig. 7:**
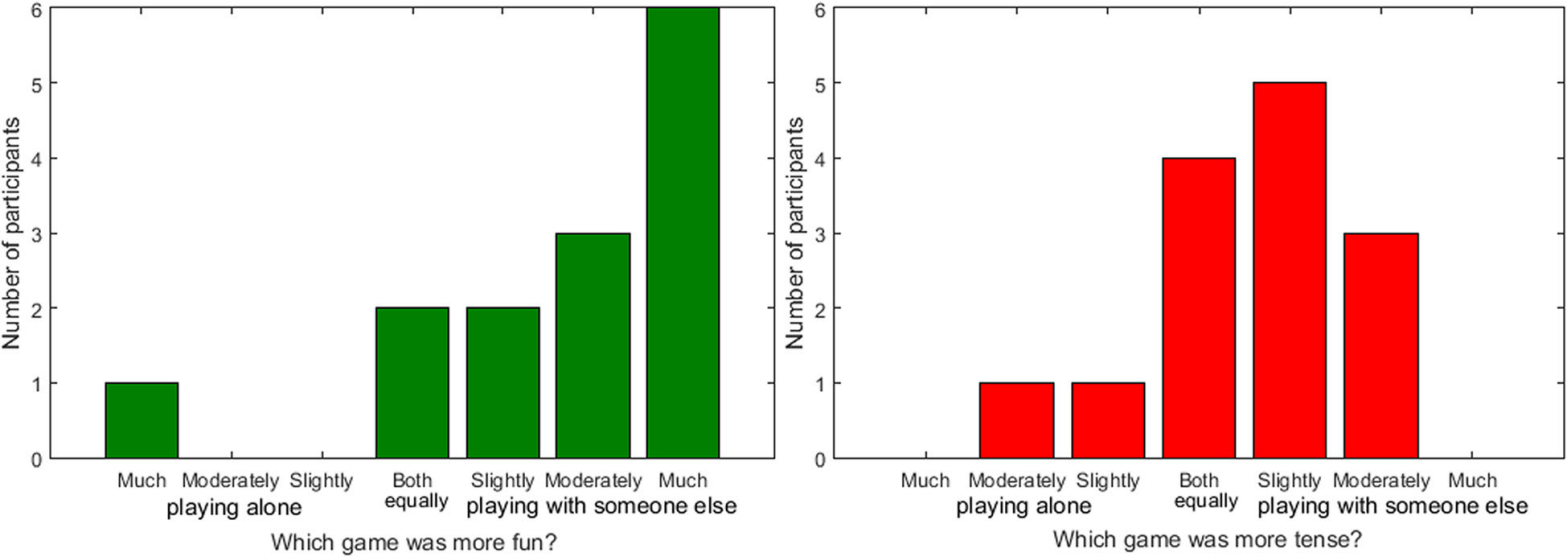
Responses of participants in the home group when asked if they found it more fun or tense to play alone or with someone else

The overall experience questionnaire first asked whether participants prefer playing alone or with someone else (i.e. their unimpaired partner). Eleven participants preferred playing with someone else, 2 had no preference and 1 preferred playing alone. More detailed results are presented in Fig. 6. In the second part of the questionnaire, participants were asked if they found it more fun or tense to play alone or with someone else. These results are presented in Fig. 7. Most participants found it more fun to play with their partner.

#### Exercise intensity and session duration

**Fig. 8:**
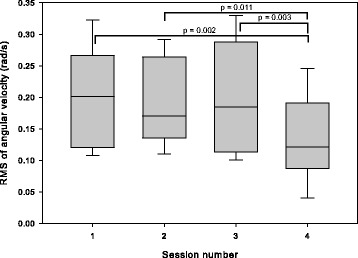
Exercise intensity calculated as the root-mean-square (RMS) of the angular velocity from the inertial sensors for participants in the home group. In sessions 1–3, participants exercised with an unimpaired partner; in session 4, they exercised alone

ANOVA found an effect of session on exercise intensity (*p* < 0.001, η^2^ = 0.35). Post-hoc tests found that exercise intensity in the 4th (SP) session was lower than in the first three (competitive) sessions, as seen in Fig. 8.

**Fig. 9:**
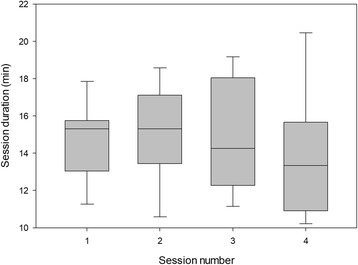
Session duration for participants in the home group. In sessions 1–3, participants exercised with an unimpaired partner; in session 4, they exercised alone

The duration of each session is presented in Fig. 9. In most cases, the game was terminated by the impaired participant due to boredom or tiredness. However, in some cases, the game was terminated by the unimpaired partner due to boredom or other obligations – this was explicitly observed in 9 sessions (involving 5 unimpaired partners) and may have affected game duration in other sessions. Finally, in 4 sessions (three competitive and one SP session), the game ended due to an external interruption such as a phone call or unexpected visitor.

#### Personality

Conscientiousness was correlated with whether participants preferred exercising alone or with someone else (ρ = −0.55, *p* = 0.039) and with whether participants found exercising alone or exercising with someone else to be more fun (ρ = −0.63, *p* = 0.016). Participants with a higher conscientiousness were more likely to prefer exercising alone and found it more fun to exercise alone.

Both conscientiousness (ρ = −0.56, *p* = 0.030) and emotional stability (ρ = −0.63, *p* = 0.011) were correlated with how often participants play computer games. Participants who played computer games more frequently had a lower conscientiousness and lower emotional stability.

One borderline significant correlation was observed: agreeableness was correlated with how often participants play computer games (ρ = −0.50, *p* = 0.056). All other correlations had *p*-values above 0.1 and are not reported.

#### Conversation level

The conversation level between participants, as rated by the experimenter on a scale from 0 to 3, was 1.7 ± 0.8 in the first session, 1.8 ± 0.9 in the second session, and 1.3 ± 1.1 in the third session.

The conversation level was correlated with pressure/tension in the first session (ρ = −0.77, *p* < 0.001), second session (ρ = −0.54, *p* = 0.038), and third session (ρ = −0.69, *p* = 0.004). The conversation level in the first session was also correlated with whether participants preferred exercising alone or with someone else (ρ = 0.88, p < 0.001) and whether participants found exercising alone or exercising with someone else to be more fun (ρ = 0.71, p = 0.004). Participants who talked to their partner more often experienced lower pressure/tension, were more likely to prefer competition, and found competition to be more fun than exercising alone.

#### Other game parameters

**Fig. 10:**
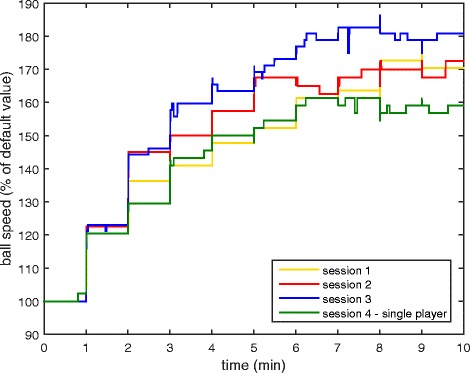
Ball speed over the first 10 min of each session, averaged across all participants in the home group

The ball speed, paddle size, and score of both players were measured throughout each session. Final values of these parameters (at the end of the session) and the maximum ball speed reached within each session are presented in Table 1. The table also includes the time in the session when participants first reached the maximum ball speed within the session. Furthermore, ball speed as a function of time is shown in Fig. 10 for the first 10 min of each session. On average, participants requested fewer than 1 manual difficulty change per session.

**Table 1:** Game parameters for each session in the home group. DF represents the default value of the parameter

Session	1.	2.	3.	4.
Final ball speed (% of DF)	157 ± 26	179 ± 21	177 ± 25	159 ± 25
Maximum ball speed reached (% of DF)	185 ± 21	185 ± 22	200 ± 23	180 ± 17
Time to first reach maximum speed (min)	8.3 ± 3.5	7.7 ± 3.1	8.9 ± 3.9	6.8 ± 3.2
Final paddle size – Impaired (% of DF)	110 ± 30	120 ± 30	110 ± 30	75 ± 25
Final paddle size – Unimpaired (% of DF)	90 ± 25	80 ± 25	90 ± 30	130 ± 25
Final score – Impaired (points)	71 ± 17	72 ± 24	69 ± 27	74 ± 26
Final score - Unimpaired (points)	80 ± 19	75 ± 15	74 ± 23	N/A

#### Experience of unimpaired partners

IMI results from unimpaired friends or relatives are presented in Table 2.

**Table 2:** Intrinsic Motivation Inventory results for unimpaired partners in the home group

Session	1.	2.	3.
Enjoyment/Interest	9.9 ± 1.2	10.2 ± 1.1	10.5 ± 1.8
Effort/Importance	10.8 ± 1.6	10.9 ± 1.7	10.5 ± 2.0
Perceived Competence	8.4 ± 1.7	9.2 ± 2.0	9.9 ± 1.9
Pressure/Tension	5.3 ± 3.4	5.6 ± 3.6	5.1 ± 3.3

The ANOVA found no differences between sessions for enjoyment/interest (*p* = 0.61, η^2^ = 0.04), effort/importance (*p* = 0.69, η^2^ = 0.06) or pressure/tension (*p* = 0.50, η^2^ = 0.05). For perceived competence, the ANOVA found an effect of session (*p* = 0.007, η^2^ = 0.32), and post-hoc tests found a difference between the 1st and 3rd session (*p* = 0.005).

### Clinical rehabilitation group

The clinical rehabilitation group consisted of 20 impaired participants who competed against each other in pairs (10 pairs) for the first three sessions. Each participant competed against a computer-controlled opponent in the fourth, SP session.

#### Intrinsic motivation inventory

Results of the IMI are presented in Table 3. ANOVA found no differences between sessions for enjoyment/interest (*p* = 0.13, χ^2^(3) = 5.64), perceived competence (*p* = 0.23, χ^2^(3) = 4.33), or pressure/tension (*p* = 0.42, χ^2^(3) = 2.85). A difference between sessions was found for effort/importance (*p* = 0.024, χ^2^(3) = 9.42), but post-hoc tests found no significant differences.

**Table 3:** Intrinsic Motivation Inventory results for the clinical group. In sessions 1–3, two participants exercised together; in session 4, each participant exercised alone

Session	1.	2.	3.	4.
Enjoyment/Interest	12.5 ± 2.0	12.0 ± 2.1	12.2 ± 2.1	12.3 ± 2.1
Effort/Importance	12.3 ± 1.6	11.6 ± 2.1	12.0 ± 2.1	12.7 ± 1.4
Perceived Competence	11.1 ± 2.0	10.4 ± 2.6	10.7 ± 2.7	11.3 ± 1.7
Pressure/Tension	6.4 ± 2.9	6.1 ± 3.4	5.8 ± 3.8	5.5 ± 3.5

#### Overall subjective experience

**Fig. 11:**
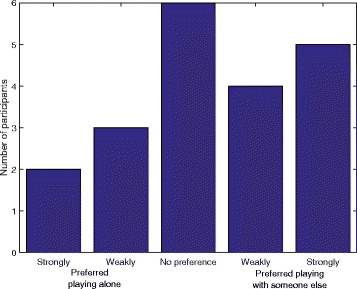
Responses of participants in the clinical group when asked if they preferred playing alone or with someone else

**Fig. 12:**
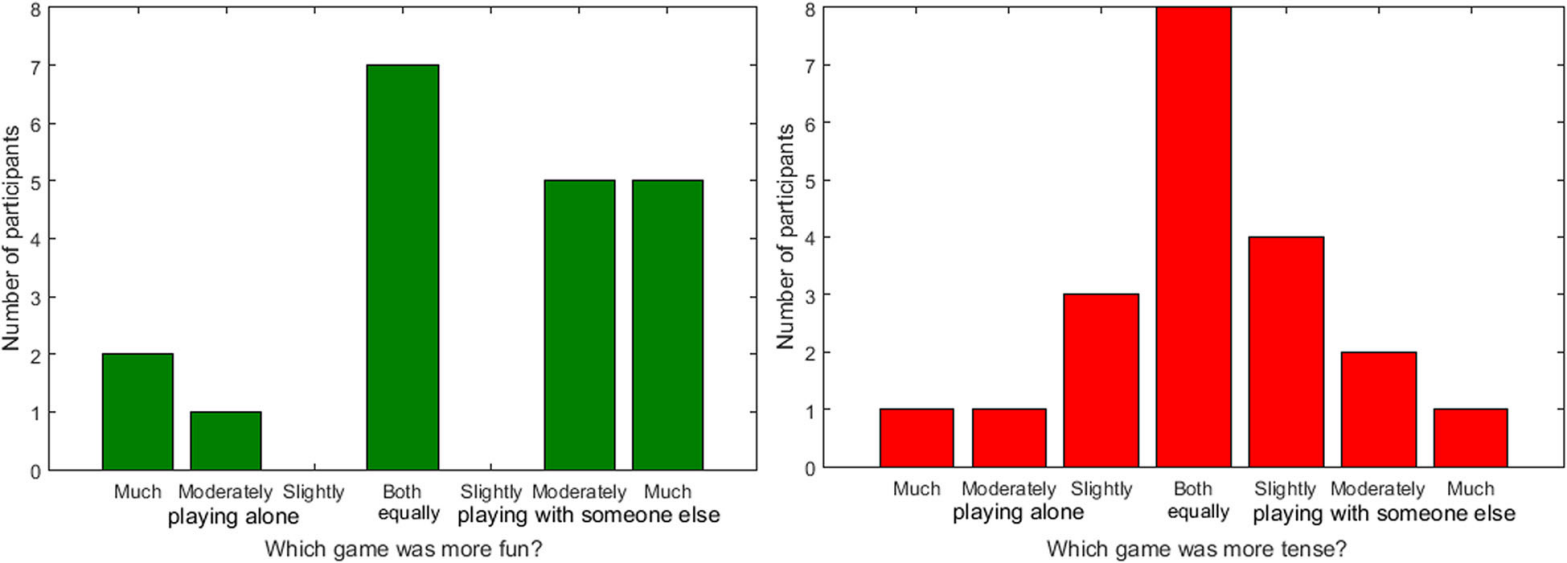
Responses of participants in the clinical group when asked if they found it more fun or tense to play alone or with someone else

The overall experience questionnaire measured whether participants prefer playing alone or with someone else (i.e. another impaired participant). Nine participants preferred playing with someone else, 6 had no preference, and 5 preferred playing alone. Detailed results are presented in Fig. 11. In the second part of the questionnaire, participants were asked if they found it more fun or tense to play alone or with someone else. These results are presented in Fig. 12.

#### Exercise intensity and session duration

Exercise intensity and session duration are presented in Table 4. There was no difference in exercise intensity between sessions (*p* = 0.48, η^2^ = 0.04). However, there was a difference in session duration between sessions (*p* = 0.010, η^2^ = 0.21). Post-hoc tests found that the second session was longer than the first and fourth sessions (both *p* < 0.05).

**Table 4:** Exercise intensity, calculated as the root-mean-square (RMS) of angular velocity from the inertial sensors, and session duration for the clinical group. In sessions 1–3, two participants exercised together; in session 4, each participant exercised alone

Session	1	2	3	4
RMS of angular velocity (rad/s)	0.180 ± 0.048	0.170 ± 0.055	0.170 ± 0.065	0.160 ± 0.055
Duration (min)	12.6 ± 1.7	14.5 ± 3.7	13.7 ± 3.0	12.8 ± 2.2

#### Personality

Agreeableness was correlated with how often participants play computer games (ρ = −0.66, *p* = 0.003). All other correlations had *p*-values above 0.1 and are not reported.

#### Conversation level

The level of conversation between participants, as rated by the experimenter on a scale from 0 to 3, was 0.9 ± 0.3 in the first session, 0.5 ± 0.5 in the second session, and 0.0 (all participants were rated a 0) in the third session. No correlations between the conversation level and other variables were found, though this is likely due to low variability: all sessions in the clinical group were rated as either a 0 or 1 with regard to conversation level.

#### Other game parameters

**Fig. 13:**
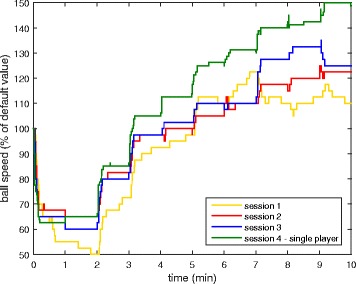
Ball speed over the first 10 min of each session, averaged across all participants in the clinical group

The ball speed, paddle size, and score of both players were measured throughout each session. Final values of these parameters (at the end of the session) and the maximum ball speed reached in the session are presented in Table 5. Furthermore, ball speed as a function of time is shown in Fig. 13 for the first 10 min of each session. On average, participants requested fewer than 1 manual difficulty change per session.

**Table 5:** Game parameters for each session in the clinical group. DF represents the default value of the parameter

Session	1.	2.	3.	4.
Final ball speed (% of DF)	125 ± 24	110 ± 24	140 ± 32	144 ± 28
Maximum ball speed reached (% of DF)	140 ± 18	145 ± 21	155 ± 33	155 ± 24
Time to first reach maximum speed (min)	7.8 ± 2.8	8.6 ± 4.3	8.3 ± 5.2	8.1 ± 2.0
Final paddle size (% of DF)	108 ± 68	103 ± 58	110 ± 85	96 ± 50
Final score (points)	62 ± 16	79 ± 25	67 ± 26	56 ± 18

## Discussion

### Home rehabilitation group

The home group shows a clear preference toward competitive exercises: enjoyment/interest and exercise intensity are both lower when exercising alone (fourth session) than when competing against an unimpaired partner (first three sessions). Furthermore, the overall experience questionnaire shows that most participants prefer exercising with their partner and find it more fun than exercising alone. Finally, there is no significant drop in enjoyment or exercise intensity from the first to the third session, indicating that novelty and habituation do not have a strong effect within three sessions.

Furthermore, the conversation level is correlated with the degree to which participants prefer competition. Although the correlation is based on subjective measures and although the cause-effect relationship is unclear, this result emphasizes the importance of social interaction in rehabilitation: patients should perform competitive exercises with a person they know and like in order to ensure an enjoyable experience. Future competitive rehabilitation games could even include elements that encourage conversation between players, potentially further increasing enjoyment.

These results indicate that competitive exercises involving a patient and their unimpaired friend/relative have the potential to improve home rehabilitation regimens by enhancing enjoyment and exercise intensity. However, this needs to be tested in a full clinical study (with 20 or more sessions) to determine whether increased engagement due to competition actually translates to better rehabilitation outcome. These next steps are discussed in the "Next steps" section.

### Why were the results of the clinical group different?

Results of the clinical group show no clear difference between competition and exercising alone, and there is thus no indication that competitive exercises performed by two patients would have an advantage in clinical rehabilitation. There are many possible reasons why results of the clinical group were not as promising as those of the home group.

First, since participants in the home group had a close relationship with their opponents (friends or relatives), they likely had a more enjoyable experience than participants in the clinical group (who competed against acquaintances from the same inpatient ward). This possibility is supported by a previous study that found that friends enjoy competitive exercises more than acquaintances [23], and by the fact that the home group exhibited higher levels of conversation than the clinical group. The social interaction may also have been enhanced by environmental factors, as participants in the home group exercised at home and thus likely felt more relaxed.

Second, participants in the home group had more experience with computer games and preferred more difficult games. Since exercising alone is essentially equivalent to the classic game of Pong, the home group may have found it more familiar and boring. Furthermore, analysis of game parameters indicated that the clinical group exhibited worse performance in the game than the home group, which may have affected enjoyment and exercise intensity.

Third, the clinical group had a significantly shorter time since injury than the home group. This has many possible consequences. For example, participants in the clinical group, who received daily motor rehabilitation sessions, may have been more tired and less interested in additional exercise than those in the home group, who mostly did not receive regular therapy. Furthermore, the clinical group may have been more focused on immediate rehabilitation goals (e.g. restoring gait and achieving independence in everyday activities) and thus less open to motivational rehabilitation games or social interaction than the home group. This is supported by the lower openness to experiences in the clinical group, though the difference was not significant (*p* = 0.080). Finally, the clinical group may have had lower cognitive abilities the home group, though this cannot be confirmed since the MMSE was only measured in the clinical group. Potential lower cognitive abilities could have also contributed to the worse in-game performance and lower conversation levels in the clinical group. Future studies should thus control for cognitive state, attitudes toward rehabilitation games, and any mental disorders (e.g. depression) that may be more common sooner after the injury.

Finally, participants in the home group may have been biased due to the recruitment method: they had to respond to a study advertisement that explicitly mentioned competitive exercises, organize a time for the sessions with the experimenter and their unimpaired partner, and allow the experimenter to visit their home. Participants willing to do this are likely predisposed toward such exercises. Conversely, participants in the clinical group were already present at the rehabilitation institute, and all scheduling was done for them, making it easier to participate. However, this confound is present in most evaluations of rehabilitation games, and removing it would require a significantly more complex study.

### Study limitations

While our results are promising, a few study limitations should be discussed. First, when exercising alone, participants actually competed against a computer-controlled opponent. This computer opponent likely behaved differently (e.g. more predictably) than a human opponent, which may have affected the results. A computer opponent with more “humanlike” behavior thus might be able to achieve the same engagement as a human opponent. We believe that this is not the case based on previous rehabilitation studies (e.g. Zimmerli et al. [14], who found no benefits to computer-controlled opponents) and general computer game research, which shows that participants enjoy competing against an opponent more if it is introduced as human rather than computer-controlled, regardless of its true nature [19, 20]. Nonetheless, future studies should consider implementing smarter or more unpredictable computer opponents.

Furthermore, the study protocol was not ideal: three sessions of competition were followed by one SP session, which may have biased the results. For example, participants may have rated the SP session lower and/or spent less time in it since they had already become used to the game. Conversely, they may have rated the SP session higher since it represented an interesting change from previous sessions. An alternative protocol would have been to include two groups: one performing only competitive exercises and one only exercising alone. Alternatively, it would be possible to include both competition and exercising alone in each session or to alternate between sessions with a partner and sessions without a partner. Still, we believe that the information obtained in our study is valid and indicates strong potential benefits of competitive exercises despite weaknesses in study design.

Finally, many of the data were obtained using subjective questionnaires. This is not only a limitation of our study – questionnaires such as the IMI have been extensively used in rehabilitation game research [6, 12, 25, 44]. Still, future studies of competitive games should utilize additional objective measures of engagement, either by analyzing in-game performance in more detail or using measurements such as electromyography and heart rate, which are common in rehabilitation games played by single patients [14] and have been used with competitive rehabilitation games in our recent pilot study [45].

### Next steps

Our study found that the benefits of competitive games are maintained over three sessions and that patients exercising with unimpaired friends or relatives at home are likely to have a more positive experience than pairs of patients exercising at a clinic. We therefore believe that such games would be most useful if provided to patients immediately upon release from inpatient rehabilitation, enhancing motivation in critical early stages of home rehabilitation. Our long-term goal is to test competitive games in a longer clinical trial (6 weeks or more) that would examine their effect on motor function using standardized clinical scales such as the Fugl-Meyer Assessment [46]. However, additional steps should be taken prior to a clinical trial.

First, our competitive game was relatively simple, with basic graphics and no long-term goals beyond scoring points. We chose such a simple game to specifically evaluate the effects of competition; however, for longer-term use, competitive rehabilitation games should be enhanced with more attractive audiovisual elements [13, 35], haptic feedback (preliminarily tested for competitive games by Baur et al. [31]) more relatable goals, and more advanced adaptation algorithms [28, 31]. Furthermore, since participants may not always want to compete and since a human co-player may not always be available, competitive games should be provided in a ‘package’ that also includes SP games and potentially even cooperative games such as the one recently tested by Mace et al. [44].

Once competitive games have been enhanced with other engaging elements and included in a package with other rehabilitation games, their effectiveness could be tested using home rehabilitation study protocols similar to the one of Nijenhuis et al. [6], who provided participants with multiple rehabilitation games in a six-week unsupervised rehabilitation regimen. Participants could be divided into two groups: an intervention group that is provided with both competitive and SP rehabilitation games, and a control group that is only provided with SP games. The amount of exercise, intensity of exercise and final rehabilitation outcome could then be compared between the two groups to determine long-term effects of including competition in a rehabilitation regimen.

## Conclusion

Competitive games played by a person with arm impairment against an unimpaired friend or relative in a home setting appear to have strong potential for arm rehabilitation, as they result in higher enjoyment and exercise intensity than exercising alone. Furthermore, enjoyment and exercise intensity did not decrease over three sessions, indicating that the benefits of competition are retained for multiple sessions. Since both enjoyment and exercise intensity are positively correlated with long-term rehabilitation outcome, competitive exercises could thus improve rehabilitation outcome compared to exercising alone. When played by two stroke survivors in a clinical setting, however, competitive games had no advantages over exercising alone. The reason for this is not clear and may be due to a combination of different factors (environment, nature of opponent, mental state etc.).

Since more promising results were obtained in home settings, we believe that competitive games would be most useful if provided to patients immediately upon release from inpatient rehabilitation, promoting engagement in critical early stages of home rehabilitation. The potential benefit of such an approach should next be validated with a full clinical trial that would examine the effect of competitive exercises played by a patient and unimpaired person on the functional outcome of home rehabilitation. If such a trial gives positive results, competitive exercises could eventually become a standard tool in motor rehabilitation, improving limb function for millions of people living with neurological injuries.

## Additional files

Additional file 1: Questionnaires used in the study. (DOCX 18 kb)

Additional file 2: Raw data from study. Includes anonymized questionnaire results, gameplay duration and exercise intensity for each participant and session. (XLSX 35 kb)

## Abbreviations

ANOVA: Analysis of variance
BBT: Box and Block Test
IMI: Intrinsic Motivation Inventory
IMU: Inertial measurement unit
MMSE: Mini-Mental State Examination
RMS: Root-mean-square
SD: Standard deviation
SP: Single-player
