# Supplementary material for: A multisession evaluation of an adaptive competitive arm rehabilitation game
Source: J Neuroeng Rehabil. 2017 Dec 6;14:128. doi: 10.1186/s12984-017-0336-9 (PMC5718145; doi:10.1186/s12984-017-0336-9)
Supplement: Supplementary file 1 — Questionnaires used in the study. (DOCX 18 kb) [file 12984_2017_336_MOESM1_ESM.docx]

**Additional file 1: Questionnaires used in study**

**PRE-GAME QUESTIONNAIRE**

1. How old are you? _______________
2. What gender are you? ________________
3. How often do you play computer games? (Choose one)

- Never
- Less than 2 hour per week
- 2-5 hours per week
- 5-10 hours per week
- More than 10 hours per week

1. How difficult do you prefer games to be? (circle one)

not difficult at all very difficult

| 1 | 2 | 3 | 4 | 5 | 6 | 7 |
| --- | --- | --- | --- | --- | --- | --- |

1. How much do you enjoy competing with other people? (circle one)

not at all very much

| 1 | 2 | 3 | 4 | 5 | 6 | 7 |
| --- | --- | --- | --- | --- | --- | --- |

**TEN ITEM PERSONALITY INVENTORY**

Here are a number of personality traits that may or may not apply to you. Please write a number next to each statement to indicated the extent to which you agree or disagree with that statement. You should rate the extent to which the pair of traits applies to you, even if one characteristic applies more strongly than the other.

I see myself as:

| not at all true very true | | | | | | | |
| --- | --- | --- | --- | --- | --- | --- | --- |
| extraverted, enthusiastic | 1 | 2 | 3 | 4 | 5 | 6 | 7 |
| critical, quarrelsome | 1 | 2 | 3 | 4 | 5 | 6 | 7 |
| dependable,  self-disciplined | 1 | 2 | 3 | 4 | 5 | 6 | 7 |
| anxious,  easily upset | 1 | 2 | 3 | 4 | 5 | 6 | 7 |
| open to new experiences, complex | 1 | 2 | 3 | 4 | 5 | 6 | 7 |
| reserved, quiet | 1 | 2 | 3 | 4 | 5 | 6 | 7 |
| sympathetic, warm | 1 | 2 | 3 | 4 | 5 | 6 | 7 |
| disorganized, careless | 1 | 2 | 3 | 4 | 5 | 6 | 7 |
| calm, emotionally stable | 1 | 2 | 3 | 4 | 5 | 6 | 7 |
| conventional, uncreative | 1 | 2 | 3 | 4 | 5 | 6 | 7 |

**INTRINSIC MOTIVATION INVENTORY**

Use this questionnaire to gauge your intrinsic motivation while playing the game. It consists of individual statements that you can agree or disagree with. There are no right and wrong answers to the questionnaire. Use the rating scale for each question to determine how much you agreed or disagreed with each statement during the game (Circle one number for each statement).

| Statement | not at all true very true | | | | | | |
| --- | --- | --- | --- | --- | --- | --- | --- |
| I put a lot of effort into the game. | 1 | 2 | 3 | 4 | 5 | 6 | 7 |
| I think I am pretty good at the game. | 1 | 2 | 3 | 4 | 5 | 6 | 7 |
| I found the game very interesting. | 1 | 2 | 3 | 4 | 5 | 6 | 7 |
| I tried as hard as I could during the game. | 1 | 2 | 3 | 4 | 5 | 6 | 7 |
| I felt very tense during the game. | 1 | 2 | 3 | 4 | 5 | 6 | 7 |
| I’m satisfied with my performance in the game. | 1 | 2 | 3 | 4 | 5 | 6 | 7 |
| I enjoyed the game very much. | 1 | 2 | 3 | 4 | 5 | 6 | 7 |
| I felt pressured during the game. | 1 | 2 | 3 | 4 | 5 | 6 | 7 |

**OVERALL EXPERIENCE QUESTIONNAIRE**

1. Which of the two game conditions did you prefer? (Choose one)

- Strongly preferred playing alone
- Weakly preferred playing alone
- No preference
- Weakly preferred playing with someone else
- Strongly preferred playing with someone else

1. Which of the two game conditions was more **fun**? (Choose one)

- Game was much more fun when playing alone
- Game was moderately more when fun playing alone
- Game was slightly more fun when playing alone
- Game was equally fun playing when alone or with someone else
- Game was slightly more fun when playing with someone else
- Game was moderately more when fun playing with someone else
- Game was much more fun when playing with someone else

1. Which of the two game conditions you felt more **tension**? (Choose one)

- Game was much more tense when playing alone
- Game was moderately more tense when playing alone
- Game was slightly more when tense playing alone
- Game was equally tense when playing alone or with someone else
- Game was slightly more tense when playing with someone else
- Game was moderately more tense when playing with someone else
- Game was much more tense when playing with someone else
